# MiR-26a Inhibits Porcine Adipogenesis by Regulating ACADM and ACSL1 Genes and Cell Cycle Progression

**DOI:** 10.3390/ani14233491

**Published:** 2024-12-03

**Authors:** Dongjie Zhang, Wanjun Hao, Rongru Zhu, Liang Wang, Xiaoxu Wu, Ming Tian, Di Liu, Xiuqin Yang

**Affiliations:** 1 Institute of Animal Husbandry, Heilongjiang Academy of Agricultural Sciences, Harbin 150086, China; djzhang8109@haas.cn (D.Z.); wlwl448@163.com (L.W.); tianming@haas.cn (M.T.); 2 College of Animal Science and Technology, Northeast Agricultural University, Harbin 150030, China; haowanjun1109@163.com (W.H.); zhurongruzi@163.com (R.Z.); wxiaox2022@163.com (X.W.)

**Keywords:** pig, adipogenesis, miR-26a, ACADM, ACSL1, RNA-seq

## Abstract

MicroRNAs play essential roles in biological processes by regulating gene expression at the post-transcriptional level. Our previous studies suggested the role of miR-26a in porcine fat accumulation. Here, through gain- and loss-of-function analyses, we first showed that miR-26a increased the proliferation of porcine preadipocytes by promoting cell division and that miR-26a inhibited the preadipocyte differentiation. Next, acyl-CoA dehydrogenase, medium chain (ACADM) was revealed to promote the proliferation and differentiation of preadipocytes for the first time. Then, it was revealed that miR-26a regulates adipogenesis by directly binding to the 3′ untranslated region of ACADM and the long-chain acyl-Co A synthetase 1 (ACSL1) gene, a previously known regulator of adipogenesis. Finally, RNA-sequencing, performed on preadipocytes overexpressing miR-26a, identified 337 differentially expressed genes in the early stage of adipogenesis; among them, nine genes were characterized as potential targets of miR-26a. The 337 genes were mainly involved in Gene Ontology terms related to cell division, indicating that cell cycle progression was also a major event regulated by miR-26a during adipogenesis. We provide novel data for understanding the molecular mechanisms underlying adipogenesis, which will contribute to controlling fat accumulation in animals.

## Simple Summary

Fat accumulation determines the lean meat percentage and meat quality directly in pigs. The clarification of mechanisms underlying fat accumulation is prerequisite for improving the traits in pigs. This study was designed to explore the mechanisms through which miR-26a regulated fat accumulation. We revealed that miR-26a promoted preadipocyte proliferation but inhibited its differentiation by directly binding to the 3′ untranslated region of ACADM and the ACSL1 gene.

## 1. Introduction

Adipose tissue is closely associated with porcine growth performance, meat production, carcass, and reproductive performance. It is also a determining factor of flavor and quality of pork, which is becoming increasingly prominent with the improvement of people’s living standard. Thereafter, adipose tissue influences final farming profits directly and is economically important in pig breeding systems [1]. Additionally, high fat intake has been extensively involved in the pathogenesis of chronic diseases, especially type 2 diabetes [2], hypertension [3], and hyperlipidemia [4], which makes consumers very concerned about fat. It is urgent for breeders to control fat deposition and distribution from a genetic perspective.

Fat accumulation is precisely organized and highly regulated. It has been demonstrated that multiple transcription factors and major genes such as CCAAT/enhancer binding protein (C/EBP), peroxisome proliferator-activated receptor gamma (PPARγ), adipose triglyceride lipase (ATGL), and lipoprotein lipase (LPL), etc., play important roles in fat formation [5,6]. Additionally, increasing evidence showed that microRNA (miRNA) repressing the expression of target mRNA by restraining its stability and/or translation [7,8] is important in the regulatory network of fat deposition.

MiRNA has been involved in every stage of fat formation, such as cell proliferation, adipogenesis, and lipogenesis. For example, the miR-17-92 cluster and miR-8 promote the differentiation of preadipocytes into adipocytes [9,10]. miR-130 suppresses adipogenesis by directly targeting PPARγ, the master regulator of adipogenesis [11]. miR-149 is associated with 3T3-L1 cell proliferation and the expression of Cyclin D19 [12]. miR-122 increases hepatic lipogenesis via suppressing the LKB1/AMPK pathway by targeting Sirt1 [13].

The MiR-26 family, composed of miR-26a and miR-26b, is highly conserved in vertebrates. MiR-26 has been shown to have bidirectional roles in regulating adipose tissue development. It is dynamically expressed in human adipocytes and promotes brown adipocyte formation via the blunting of ADAM17 activity [14]. MiR-26 promotes triacylglycerol synthesis via targeting insulin-induced gene 1 in goat mammary epithelial cells [15]. Studies also showed a negative role of miR-26a in lipid metabolism in that miR-26 was downregulated in overweight humans and obese mouse models, and the silencing of endogenous miR-26a increased fatty acid synthesis in mice [16]. Additionally mouse miR-26 inhibits adipocyte progenitor differentiation and fat formation by targeting Fbxl19 [17]. These indicate that the role of miR-26 in fat formation is complicated and distinct among species, even among cells from different origins of the same animal. In pigs, many efforts are needed to clarify the role of miR-26a in fat accumulation. The acyl-CoA dehydrogenase (ACAD) family is involved in the oxidation of fatty acids by converting acyl-CoA into acetyl-CoA [18]. As an enzyme in fat metabolism, the importance of ACADM cannot be overstated. However, its effects on the proliferation and differentiation of preadipocytes have not been described yet. This study was designed to analyze the role of miR-26a in the proliferation and differentiation of porcine preadipocytes, based on which the target genes were identified with a dual-luciferase reporter system. Furthermore, RNA sequencing (RNA-seq) was performed to further explore target genes and mechanisms through which miR-26a regulates preadipocyte differentiation at the early stage of induction. Pigs are closely related to human beings in terms of genetics, physiology, and anatomy. The results will provide data for further understanding the mechanisms underlying the regulatory network of miR-26a in fat deposition, which will contribute to controlling fat accumulation in humans.

## 2. Materials and Methods

### 2.1. Ethics Statement

All methods are reported in accordance with ARRIVE guidelines (https://arriveguidelines.org/arrive-guidelines; accessed on 1 March 2024). Animals were treated strictly according to the protocol provided by the Laboratory Animal Welfare and Ethics Committee of Northeast Agricultural University (NEAUEC20240288).

### 2.2. Animals, Tissues, and cDNA

Min pigs at the age of 30 d old were obtained from the Institute of Animal Husbandry, Heilongjiang Academy of Agricultural Sciences, Harbin, China. Fat tissues were collected immediately after the pigs were slaughtered. Samples for RNA isolation were stored at −80 °C. RNA was isolated using a Trizol reagent (Invitrogen, Carlsbad, CA, USA). cDNA was synthesized by using the HiScript III 1st Strand cDNA Synthesis Kit (+gDNA wiper) (Vazyme, Nanjing, China).

### 2.3. Plasmids and Oligonucleotide Sequences

The complete coding sequence of the porcine ACADM gene was amplified and inserted into pCMV-HA at enzyme sites EcoRI and KpnI. Reporter genes containing the 3′ UTR of ACADM and the ACSL1 gene, respectively, were constructed with the psiCHECKTM-2 backbone using the XhoI enzyme. Site-directed mutation was performed with an overlap extension PCR as described previously [19]. miRNA mimics, inhibitors, and siRNAs against ACADM were synthesized by General Biotechnology (Hefei, China). Primers were designed using primer premier 5.0 software and synthesized by Genesoul technology (Harbin, China). Plasmids overexpressing and siRNAs against ACSL1 were obtained previously [20]. Primer and nucleotide sequences used here are given in Supplementary Table S1.

### 2.4. Real-Time Quantitative PCR

The real-time quantitative PCR (qPCR) was carried out with the ChamQ Universal SYBR qPCR Master Mix (Vazyme, Nanjing, China). The qPCR was performed in a final volume of 10 µL containing 0.2 µmol/L of the sense and antisense primers, a 2 × ChamQ SYBR qPCR Master Mix (5 µL), 50 × ROX Reference Dye 1 (0.2 µL), template cDNA (0.5 µg). The thermal profiles were 95 °C (30 s), 40 cycles of 95 °C (10 s), and 60 °C (30 s). The data were calculated with the 2−ΔΔCt method, and β-actin was selected as a reference. Primer sequences for the qPCR are given in Supplementary Table S1.

### 2.5. Cell Culture and Transfection

Porcine primary preadipocytes were cultured as described previously [21]. Briefly, preadipocytes were obtained from backfat tissues of 30-day-old Min pigs. After the pigs were slaughtered, all the backfat were collected. The tissues were washed with sterile phosphate-buffered saline (PBS). The connective tissues and contaminated muscle were removed carefully. Afterwards, the fat tissues were washed in PBS supplemented with 1% penicillin–streptomycin (Invitrogen) three times and cut into small pieces. The tissues were digested using 0.1% collagenase I (Invitrogen) at 37 °C for 40–50 min, mixed with equal volumes of the medium containing 1% penicillin–streptomycin and 10% fetal bovine serum (FBS; Gibco, Carlsbad, CA, USA). The mixture was filtered through 400-mesh filters and centrifuged for 5 min at 1000 rpm. The cells were resuspended in Dulbecco’s modified Eagle’s medium/Nutrient Mixture F-12 (DMEM/F12) supplemented with 1% penicillin–streptomycin (Invitrogen) and 10% FBS (Gibco). The medium was changed every 48 h.

Preadipocyte differentiation was induced with 0.5 mmol/L of 3-isobutyl-1-methylxanthine, 1 µmol/L of dexamethasone, and 5 µg/mL of insulin for 2 days, and then 5 µg/mL of insulin was used to maintain the differentiation until the cells were collected. PK-15 cells were cultured as described previously [20]. Transient transfection was carried out using the Lipofectamine 2000 reagent (Invitrogen) according to the manufacturer’s protocol. Briefly, 5 µL (50 nmol/L) of oligo nucleotide and 7.5 µL of Lipofectamine 2000 were first diluted in 100 µL of DMEM, respectively. The two mediums were then mixed and stored at room temperature for 15 min. Finally, the products were added into six-well plates. For cells cultured in other type plates, the volume of oligo nucleotide and Lipofectamine 2000 was increased/decreased proportionally. Plasmid DNA and Lipofectamine 2000 were used in a 1:3 ratio. As for cotransfection, a 1:1 mixture of plasmid DNA and oligo nucleotide was used.

### 2.6. Oil Red O Staining and Quantitative Analysis

At 24 h post-transfection, the preadipocytes were induced to differentiate for eight days. Then, the cells were stained with Oil Red O kit (Leagene, Beijing, China) and photographed with a light microscope (Carl Zeiss AG, Jena, Germany). After being isolated with isopropanol, cellular Oil Red O was quantified with optical absorbance at 510 nm.

### 2.7. Luciferase Reporter Gene Analysis

When PK-15 cells were cultured to 70–80% confluence, each of the reporter genes, including the wild type and mutant type, was transfected together with miR-26a mimics. At 24 h post-transfection, the cells were collected, and luciferase activities were measured with the Dual Luciferase Reporter Assay Kit (Vazyme). The relative luciferase activities were calculated as a ratio of Renilla to firefly luciferase value.

### 2.8. Cell Counting Kit 8 Assay

Preadipocytes were seeded in 96-well plates at a density of 3000. At 30% confluence, the cells were transfected with Lipofectamin 2000 (Invitrogen). Cells were subjected to a Cell Counting Kit-8 (CCK-8) (Beyotime, Shanghai, China) assay at 0, 12, 24, 36, and 48 h, according to the manufacturer’s instructions using a Tecan Microplate Reader Infinite F50 (Tean GENios, Mannendorf, Switzerland).

### 2.9. 5-Ethynyl-2′-deoxyuridine Incorporation Assay

Preadipocytes were seeded in 96-well plates at a density of 3000, cultured for 16 h, and then transfected as described above. At 24 h post-transfection, the cells were subjected to a 5-ethynyl-2′-deoxyuridine (EdU) incorporation assay with a BeyoClick™ EdU-555 kit (Beyotime). The cells were observed using an Olympus inverted fluorescence microscope IX71 (Olympus, Tokyo, Japan).

### 2.10. Flow Cytometry Analysis

Preadipocytes were cultured in six-well plates and transfected with Lipofectamin 2000 (Invitrogen). At 24 h post-transfection, the cells were digested with trypsin for 1 min after being washed with PBS. The cells were then collected and stained by using a cell cycle staining Kit (MultiSciences, Hangzhou, China). The cell cycle was analyzed with the Aglient Novo Cyte (Aglient; Palo Alto, CA, USA) Flow Cytometer.

### 2.11. RNA Sequencing and Data Analysis

At a confluence of 70–80%, the preadipocytes were transfected with miR-26a mimics and negative control (NC) sequences, respectively, and cultured for 48 h. The cells were then induced to differentiate as described above. At 48 h post-induction, the cells were subjected to RNA sequencing by Biomark technologies (Beijing, China) on a NovaSeq6000 sequence analyzer (Illumina, San Diego, CA, USA) to identify genes regulated by miR-26a at the early stage of adipogenesis.

The raw data were first cleaned with SOAPnuke software (v2.1.0) [22] with default parameters. The clean data were mapped to the porcine reference genome (Sscrofa11.1_release109) using HISAT2 (v2.1.0) [23]. The unique mapped reads were assembled using StringTie software (v2.1.7) [24]. The reads with transcripts per million > 1 in at least half of the samples were used for further analysis. The expression level of genes was quantified with fragments per kilobase million (FPKM) by Cufflinks (v2.2.1) [25]. DEGs between two groups were characterized using DESeq2 (v1.22.2) [23] with the criteria of fold change (FC) ≥ 1.5 and *p* < 0.05. The DEGs were enriched with the GO program to analyze their roles as described previously [21].

### 2.12. Statistical Analysis

All experiments were conducted three independent times, each with triplicates. Data were presented with mean ± standard deviation. GraphPad Prism (version 9.5.1; GraphPad, San Deigo, CA, USA) was used to process the data. Differences between the two groups were compared with an unpaired *t*-test, while those among multiple groups were compared with one-way ANNOVA. * indicates *p* < 0.05; ** indicates *p* < 0.01.

## 3. Results

### 3.1. MiR-26a Promotes Proliferation of Porcine Preadipocytes

**Figure 1:**
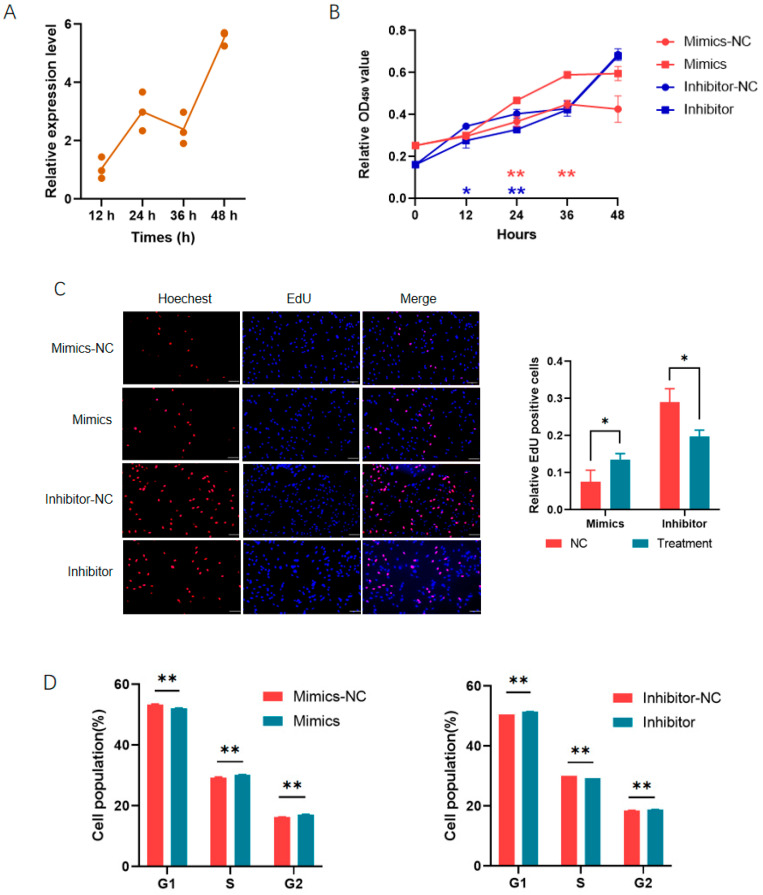
MiR-26a promoted the proliferation of porcine preadipocytes: (**A**) The expression profile of miR-26a during preadipocyte proliferation. The value at 12 h was used as 1; (**B**,**C**) MiR-26a promoted preadipocyte proliferation as revealed by the CCK-8 assay (**B**) and EdU staining (**C**). The bar is 100 μm, the same as below; (**D**) MiR-26a promoted the cell cycle progression. *, *p* < 0.05; **, *p* < 0.01.

We first analyzed the role of miR-26a in the proliferation of porcine preadipocytes. During preadipocyte proliferation, the expression of miR-26a increases gradually (Figure 1A). The cell viability was increased by miR-26a mimics while decreased by the inhibitor in porcine preadipocytes, as revealed by the CCK-8 assay (Figure 1B). EdU analyses further confirmed the positive role of miR-26a in preadipocyte proliferation as the up-regulation of miR-26a promoted the proliferation, while the inhibition of miR-26a repressed it (Figure 1C). Flow cytometry analyses showed that miR-26a mimics decreased the cell population in the G0/G1 phase and increased it in the S and G2/M phase; at the same time, opposite results were obtained when the preadipocytes were treated with the inhibitor (Figure 1D). The results indicated that miR-26a increased preadipocyte proliferation by promoting the cell cycle progression.

### 3.2. MiR-26a Inhibits Differentiation of Porcine Preadipocytes

**Figure 2:**
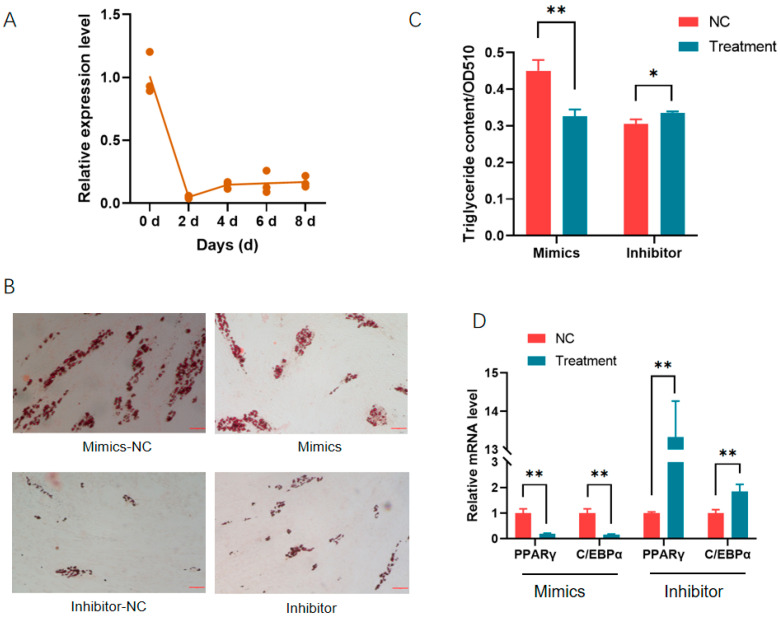
MiR-26a inhibited the differentiation of porcine preadipocytes: (**A**) The expression profile of miR-26a during preadipocyte differentiation. The value at 0 d was used as 1; (**B**,**C**) MiR-26a inhibited the formation of droplets as revealed by Oil O staining (**B**) and extraction assays (**C**) at eight days post-induction. The bar is 100 μm, the same as below; (**D**) MiR-26a repressed the expression of adipogenic markers PPARγ and C/EBPα. *, *p* < 0.05; **, *p* < 0.01.

During preadipocyte differentiation, the expression of miR-26a decreased sharply at 2 d and then kept constant until 8 d post-induction (Figure 2A). Oil Red O staining showed that miR-26a inhibited the differentiation of preadiopocytes as the mimics decreased the formation of fat droplets (*p* < 0.01), while the inhibitor increased it (*p* < 0.01) (Figure 2B,C). At the same time, the expression of the marker genes of preadipocyte differentiation, PPARγ and C/EBPα, was measured at six days post-induction. The overexpression of miR-26a repressed, while the inhibition of miR-26a promoted, their expression significantly (*p* < 0.01; Figure 2D).

### 3.3. MiR-26a Regulates Adipogenesis by Targeting ACADM

**Figure 3:**
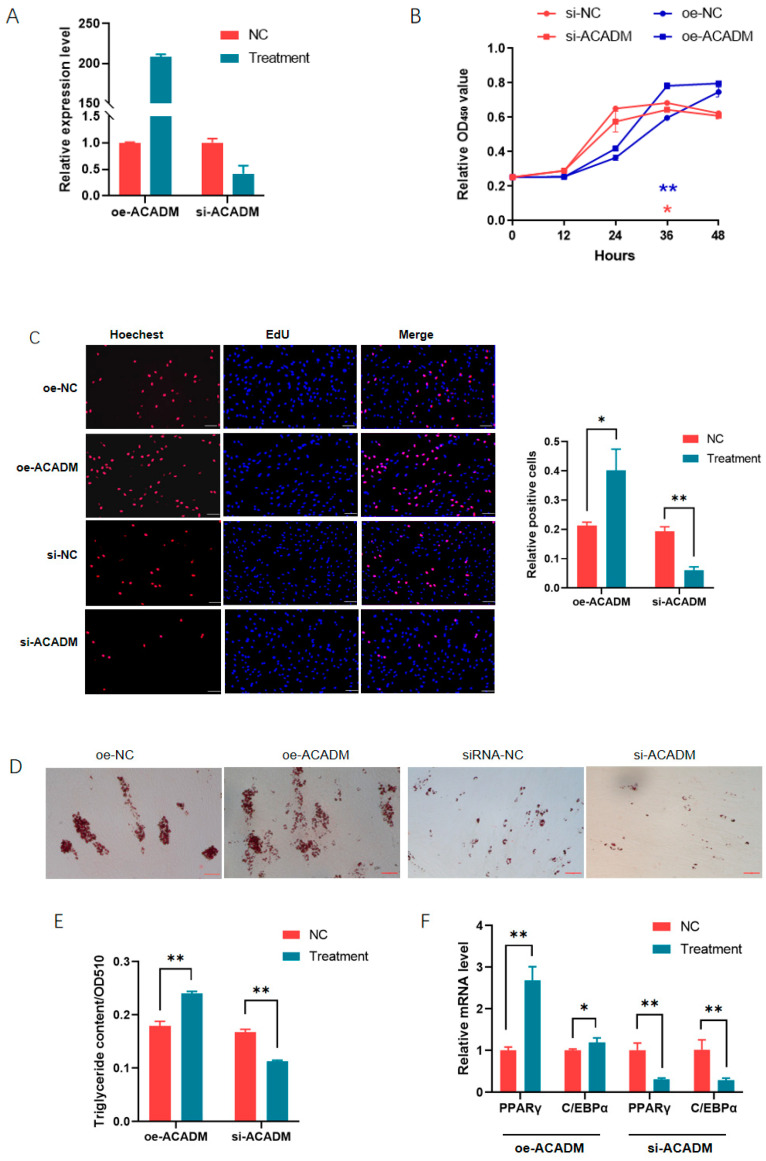
ACADM the promoted proliferation and differentiation of porcine preadipocytes: (**A**) Efficiencies of plasmids overexpressing and siRNA against the ACADM gene; (**B**,**C**) ACADM promoted the cell proliferation as revealed by the CCK-8 assay (**B**) and EdU staining (**C**); (**D**,**E**) ACADM promoted the formation of droplets as revealed by Oil O staining (**D**) and extraction assays (**E**); and (**F**) ACADM enhanced the expression of adipogenic markers PPARγ and C/EBPα. *, *p* < 0.05; **, *p* < 0.01.

To reveal the mechanisms underlying the regulation of miR-26a in fat deposition, target genes were predicted by using TargetScan, TarBase, and the miRDB program, and ACADM was found to be a credible target. We first analyzed the role of ACADM in the proliferation and differentiation of porcine preadipocytes. Plasmids overexpressing and siRNA against ACADM were obtained successfully (Figure 3A). Through the CCK-8 and EdU assay, we made clear that ACADM promoted the preadipocyte proliferation (Figure 3B,C). Oil Red O staining and extraction assays showed that ectopic ACADM promoted preadipocyte differentiation, while knocking down ACADM inhibited it (Figure 3D,E). The expression of PPARγ and C/EBPα in response to ACADM treatment at six days post-induction further confirmed that ACADM promoted adipogenesis (Figure 3F).

**Figure 4:**
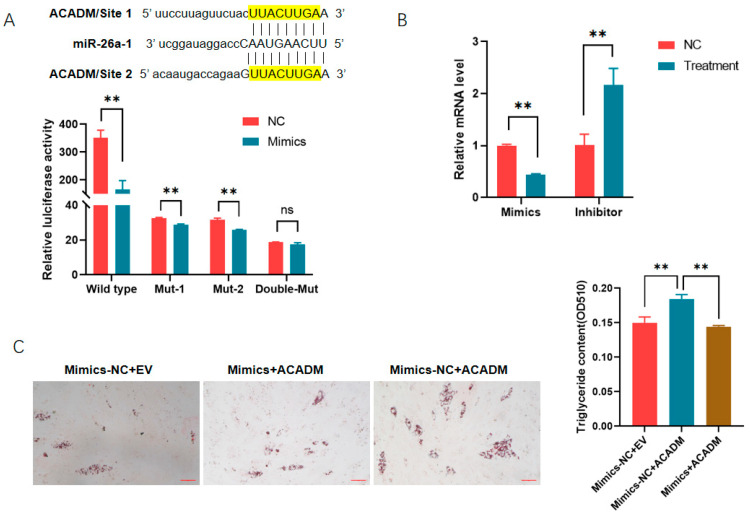
MiR-26a regulates adipogenesis by targeting ACADM: (**A**) miR-26a inhibited the expression of ACADM by targeting the 3′ UTR as revealed by reporter gene analysis; (**B**) MiR-26a inhibited the mRNA level of ACADM in PK-15 cells; and (**C**) MiR-26a reversed the promoting effects of ACADM on adipogenesis. **, *p* < 0.01; ns = not significant.

Next, the regulatory relationship between miR-26a and ACADM was analyzed. Two closely spaced binding sites for miR-26a were predicted in the 3′ UTR. MiR-26a mimics decreased the activities of the reporter gene containing the ACADM 3′ UTR (*p* < 0.01) in PK-15 cells. The single deletion of any of the sites did not influence the inhibitory effects of mimics on the reporter genes, while double deletion of the sites abolished the repression of mimics, indicating that both sites are active for ACADM binding (Figure 4A). Additionally, the mRNA level of ACADM was significantly decreased by miR-26a mimics (*p* < 0.01) and increased significantly by the inhibitor (*p* < 0.01) in PK-15 cells (Figure 4B). Considering the fact that miR-26a inhibited adipogenesis while ACADM promoted it, a regulatory axis might exist between them during adipogenesis. Thereafter, rescue experiments were performed, and the results showed that the promoting effects of ACADM on adipogenesis were reversed by miR-26a overexpression (Figure 4C), making clear that miR-26a inhibited the adipogenesis by targeting the ACADM gene.

### 3.4. MiR-26a Is Also a Regulator of ACSL1 During Adipogenesis

**Figure 5:**
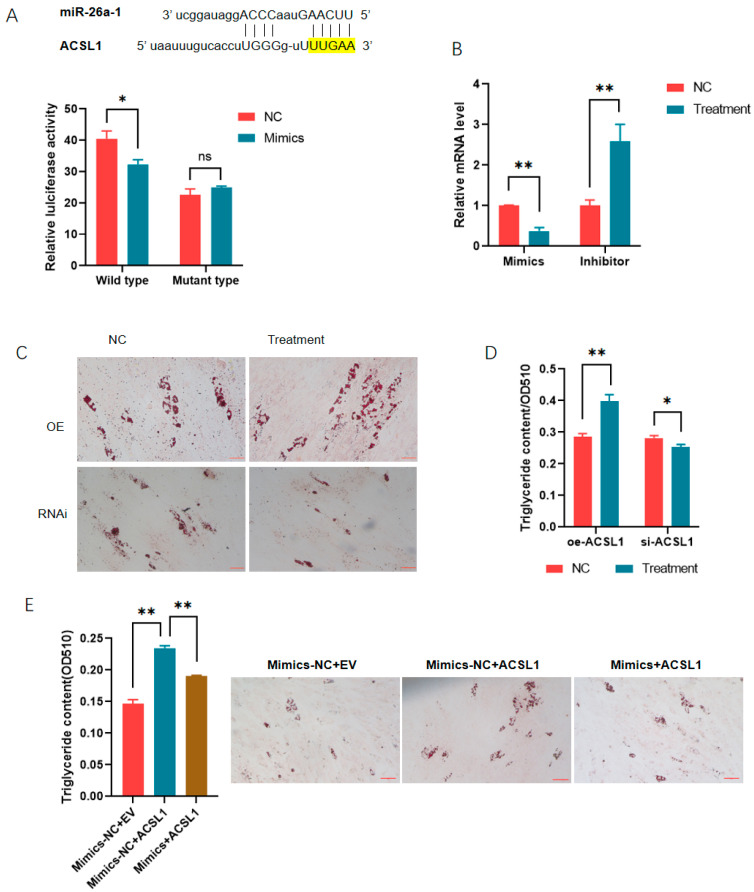
MiR-26a regulated the role of ACSL1 during adipogenesis: (**A**) miR-26a inhibited the expression of *ACSL1* by targeting the 3′ UTR; (**B**) MiR-26a inhibited the mRNA level of *ACSL1* in PK-15 cells; (**C**,**D**) ACSL1 promoted the differentiation of preadipocytes as revealed by Oil O staining (**C**) and extraction assays (**D**); and (**E**) MiR-26a reversed the promoting effects of ACSL1 on adipogenesis. *, *p* < 0.05; **, *p* < 0.01; ns = not significant.

Through bioinformatic analysis, we found that there was also one putative binding site for miR-26a in the 3′ UTR of long-chain acyl-Co A synthetase 1 (ACSL1). ACSL1 has been revealed to promote adipogenesis in Min pigs. The role of miR-26a in adipogenesis regulated by ACSL1 was analyzed. Reporter gene analysis showed that the overexpression of miR-26a inhibited the activities of the ACSL1 3′ UTR, while it had no effects on 3′ UTR mutants absent from the binding site (Figure 5A). The mRNA level of ACSL1 was decreased significantly by miR-26a mimics and increased significantly by the inhibitor in PK-15 cells (Figure 5B). Then, rescue experiments showed that the promoting effects of ACSL1 on adipogenesis were reversed by miR-26a mimics (Figure 5C–E).

### 3.5. RNA-Seq Reveals Mechanisms Through Which miR-26a Regulates Adipogenesis

**Figure 6:**
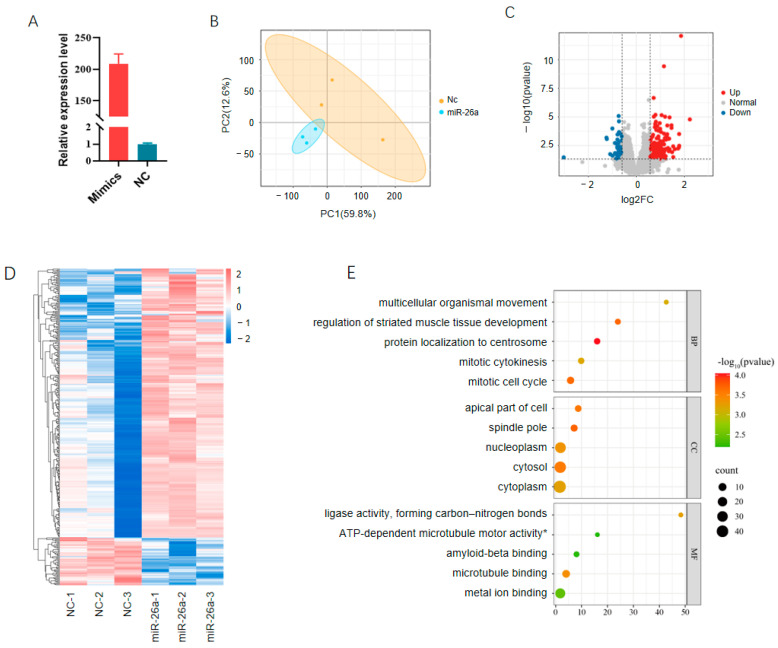
RNA-seq reveals cell cycle progression is important for adipogenesis: (**A**) Overexpressing efficiencies of mimics at the time of RNA-seq; (**B**) principal component analysis plot of RNA-seq data; (**C**) volcano plot of DEGs; (**D**) heatmap of DEGs; and (**E**) top 5 GO terms enriched by DEGs in each category.

To further analyze the mechanisms through which miR-26a suppresses porcine adipogenesis, we performed RNA-seq on preadipocytes transfected with mimics or NC sequences at the early stage of differentiation. A total of 119,975,614 clean reads were obtained with an average of 19,995,935 per sample (Supplementary Table S2). Principal component analysis (PCA) showed that NC and treatment groups separated well, indicating a good quality of RNA-seq (Figure 6A,B). A total of 337 differentially expressed genes (DEGs), including 286 upregulated and 51 downregulated, were identified in preadipocytes overexpressing miR-26a compared to NC groups (Figure 6C,D; Supplementary Table S3). DEGs were enriched in all three Gene Ontology (GO) categories, including the biological pathway (BP), the cellular component (CC), and the molecular function (MF), and enriched GO terms were mainly involved in cell cycle progression (Figure 6E).

From down-regulated DEGs, we sought putative targets of miR-26a using TargetScan, TarBase, and miRDB. A total of nine genes, including the SRY-box containing gene17 (SOX17), the RCSD domain containing 1 (RCSD1), dual-specificity phosphatase 2 (DUSP2), semaphorin 6A, sorting nexin 15 (SNX15), zinc finger, and BTB domain containing 42 (ZBTB4), frequently rearranged in advanced T-cell lymphomas 1 (FRAT1), short-chain dehydrogenase/reductase member 3 (DHRS3), and zinc-finger SWIM domain-containing protein (ZSWIM), were predicted to have multiple binding sites by at least one of the three algorithms (Supplementary Table S4).

## 4. Discussion

Since discovery, miRNAs have attracted widespread attention from people owing to the mechanisms by which they work. Through binding to target genes, a specific miRNA can regulate numerous genes directly, thus playing important roles in various cellular processes such as cell proliferation, apoptosis, differentiation, and migration [26]. It is important for us to reveal the role of miRNA in fat deposition, which will contribute to controlling fat formation in mammals. Here, we made clear that miR-26a promoted proliferation and inhibited the differentiation of porcine primary preadipocytes, and two genes, involved in fat mechanisms, were demonstrated to be targeted by miR-26a during adipogenesis. Additionally, RNA-seq reveals that cell cycle progression might be a major event affected by miR-26a during its regulation of early adipogenesis. The results will provide a basis for comprehensively revealing the role of miR-26a in fat production.

The MiR-26 family has been extensively involved in tumorigenesis by targeting critical regulators of the cell cycle [27], cell differentiation [28], and development [29]. Also, a few studies showed the effects of miR-26 on fat production with diverse results, and, during preadipocyte differentiation, the role changes with induction time, cell origin, and species [14,15,16,17]. These indicate that more efforts need to be made to clarify the role of miR-26 in fat production. Here, miR-26a was selected as a candidate for fat deposition based on our previous research.

As expected, porcine miR-26a plays a role in preadipocyte proliferation and differentiation as revealed by gain- and loss-of-function assays. Researchers are more concerned about the effects of miR-26a on cell differentiation in adipocyte biology. Little was known on its role in preadipocyte proliferation. Fat accumulation results from increases in adipocyte size (hypertrophy) and/or number (hyperplasia) [30,31]; thereafter, preadipocyte differentiation and proliferation are both important for fat accumulation. Here, we made clear for the first time that miR-26a promoted the proliferation of porcine preadipocytes obtained from subcutaneous adipose tissue. The effects of miR-26a on adipogenesis in porcine preadipocytes contradict those from human cells, including mesenchymal stem/stromal cells [32] and multipotent adipose-derived stem cells [14], while consistent to that from mouse stromal vascular fraction cells [17]. The results further confirmed that the regulatory role of miR-26a is complicated and cell-specific.

ACADM is the most important member in the family and catalyzes the initial step of dehydrogenation in the β-oxidation of medium-chain fatty acids (MCFAs) [33]. It is known that triglyceride, composed of glycerol and three fatty acids, is classified into short-, medium-, and long-chain triglycerides (SCTs, MCTs, and LCTs) according to the chain length of fatty acids. Adipose tissues are mainly composed of LCTs, while MCTs look more like glucose than fat [34,35]. Compared to LCFAs (long-chain fatty acids) or LCTs, MCFAs or MCTs contribute to lowering triglyceride accumulation in chicken hepatocytes [36] and in rat livers [37,38] and to decreasing body weight and fat mass as well [39,40]. It was further revealed that MCFA inhibited lipid deposition by regulating key lipid-sensing genes, including fatty acid synthase, hormone-sensitive lipase, and lipoprotein lipase, etc., in human liver cells [15]. These results indicate that MCFAs/MCTs are negative regulators of triglyceride accumulation, which makes MCT a favorable diet over LCT to avoid various chronic diseases associated with fat [41]. From this point of view, we speculated that ACADM, an enzyme primarily responsible for decomposing MCFA, might promote triglyceride mass. As expected, through gain- and loss-of-function analyses, we confirmed that ACADM promoted lipid droplet formation during preadipocyte differentiation. ACADM has been extensively involved in the pathogenesis of diseases, especially in cancer, owing to its importance in the β-oxidation of MCFA [42,43,44]. However, little was known on the role of ACADM in adipogenesis. The results presented here identified a previously unknown role of ACADM and suggested a potential of ACADM for manipulating fat deposition in mammals.

We characterized 337 genes regulated by miR-26a during the early adipogenesis of porcine preadipocytes with RNA-seq. Through Gene Set Enrichment Analyses, we found miR-26a posed an important effect on mitosis during its regulation of adipogenesis. Cell cycle arrest is as important as the induction of the expression of PPARγ and C/EBPα during the adipogenic differentiation process. These two events lead to the accumulation of fat droplets in cytosol jointly [45,46]. PPARγ activation is tightly inherent with the induction of adipogenic specific genes and exit from the cell cycle [45]. Consistently, we showed that, in undifferentiated preadipocytes, miR-26a increased proliferation by promoting cell cycle progression. The results indicated that cell division is an important step for the regulation of miR-26a on fat accumulation. However, the expression of PPARγ and C/EBPα was not changed by miR-26a, as revealed by RNA-seq. The phenomenon might be caused by the time point detected because only cells in the early stage of differentiation were selected for RNA-seq. That is also the reason why ACADM and ACSL1 were not found among DEGs.

A total of nine genes were identified as potential targets of miR-26a through the combined analyses of RNA-seq data and bioinformatic prediction. None of these genes to our knowledge have been involved in adipogenesis except for DUSP2. DUSPs are a subgroup of protein tyrosine phosphatases that suppress mitogen-activated protein kinase (MAPK) activity, thus playing a central role in regulating MAPK-dependent biological processes. It has been demonstrated that the MAPK signal transduction cascade is essential for adipogenesis [47,48,49]. DUSP1 and -2 have been shown to inhibit MAPK activity and adipogenesis at the early stage of differentiation in mice [50]. DUSP2 was also revealed to be a candidate for fat deposition through profiling mRNA and miRNA in backfat tissues of pigs with differential backfat thickness by RNA-seq [51]. Both FRAT1 and SOX17 are involved in canonical Wnt signaling, an important pathway regulating adipogenesis [52]. FRAT1 is a positive regulator of the Wnt/beta-catenin pathway [53,54]. SOX17 interacts with the canonical Wnt signaling pathway through co-occupying Wnt-responsive enhancers with β-catenin to specify and pattern the endoderm fate and thus might affect the recruitment of β-catenin to chromatin to control Wnt-responsive transcription across many biological contexts [55]. These results indicated the inherent relationship between miR-26a, genes, and adipogenesis.

## 5. Conclusions

In this study, the role and the underlying mechanisms of miR-26a on porcine adipogenesis were analyzed. We first made clear that miR-26a increased the proliferation of preadipocytes by promoting cell cycle progression. Next, it was demonstrated that miR-26a inhibited adipogenesis by directly targeting the 3′UTR of ACADM and ACSL1. Then, hundreds of genes regulated by miR-26a were identified with RNA-seq in the early stage of adipogenesis; among them, nine were identified as potential targets. Finally, it was found that DEGs were mainly involved in GO terms related to cell division, indicating that cell cycle progression was also one of the major events regulated by miR-26a in adipogenesis. The data obtained contribute to understanding the molecular mechanisms underlying fat accumulation in pigs.

## Data Availability

The data presented in this study are openly available in the database BIG Submission (https://ngdc.cncb.ac.cn/gsub/, Project: PRJCA029950, accessed on 1 September 2024).

## Supplementary Materials

The following supporting information can be downloaded at https://www.mdpi.com/article/10.3390/ani14233491/s1, Table S1: Primer and oligonucleotide sequences; Table S2: Statistics of RNA-seq; Table S3: Differentially expressed genes; and Table S4: Potential targets of miR-26a identified.
