# Supplementary material for: MiR-26a Inhibits Porcine Adipogenesis by Regulating ACADM and ACSL1 Genes and Cell Cycle Progression
Source: Animals (Basel). 2024 Dec 3;14(23):3491. doi: 10.3390/ani14233491 (PMC11640176; doi:10.3390/ani14233491)
Supplement: Supplementary file 1 [file animals-14-03491-s001.zip › Table S1 Primer and oligonucleotide sequences.pdf]

Table S1-1 Primers used in the study

| Names | Sequences (5'-3') | Purpose |
|---|---|---|
| ACADM-CDS-F | CGGAATTCTTATGGCAGCGATGTTTAGG | CDS amplification |
| ACADM-CDS-R | GGGGTACCTTAATTTTTATACCGGCCAA | |
| ACADM-F | GATGAAGCTACCAAGTATGC | ACADM quantification |
| ACADM-R | CATAATAGGTATTTCGGCG | |
| ACADM-WT-F | CCGCTCGAGTCTATACTAAACCTATGCAACAG | Amplification of 3' UTR of ACADM |
| ACADM-WT-R | ATTTGCGGCCGCTACAATGTATCCACATGAAATG | |
| ACADM-miR-26a-1-MUT-F1 | CCTTAGTTCTACATTTCATTAAC | Deletion of binding site 1 of miR-26a-1 in the 3' UTR of ACADM |
| ACADM-miR-26a-1-MUT-R1 | GTTAATGAAATGTAGAACTAAGG | |
| ACADM-miR-26a-1-MUT-F2 | ATGACCAGAAGAAATCTTTTT | Deletion of binding site 2 of miR-26a-1 in the 3' UTR of ACADM |
| ACADM-miR-26a-1-MUT-R2 | AAAAAGATTTCTTCTGGTCAT | |
| ACSL1-F | GTAGTGAGCGATTGTTCAGCGTTTG | ACSL1 quantification |
| ACSL1-R | GCGAGAGGCAAGAAAGAGATCAGAG | |
| ACSL1-WT-F | CCGCTCGAGACCTTCCCCATTTCACACTC | Amplification of 3' UTR of ACSL1 |
| ACSL1-WT-R | ATTTGCGGCCGCGCAAACATTCAAAACCCCA | |
| ACSL1-miR-26a-1-MUT-R | ATTTGCGGCCGCCCAAGGTGACAAATTATTGAC | Deletion of binding site 1 of miR-26a-1 in the 3' UTR of ACSL1 |
| C/EBPα-F | TAGACAAGAACAGCAACGAG | C/EBPα quantification |
| C/EBPα-R | ACCTTCTGTTGAGTCTCCACG | |
| PPARγ-F | CATTCGCATCTTTCAGGG | PPARγ quantification |
| PPARγ-R | GGACGCCATACTTTAGG | |
| GAPDH-F | GGTGAAGGTCGGAGTGAACG | Reference for mRNA quantification |
| GAPDH-R | CTCGCTCCTGGAAGATGGTG | |
| U6-F | CTCGCTTCGGCAGCACA | Reference for miRNA quantification |
| U6-R | AACGCTTCACGAATTTGCGT | |
| URP | TGGTGTCGTGGAGTCG | Reverse primer of miRNA quantification |
| miR-26a-1-F | ACACTCCAGCTGGGTTCAAGTAACCCAGGA | Forward primer of miR-26a-1 quantification |
| miR-26a-1-RT | CTCAACTGGTGTCGTGGAGTCGGCAATTCAGTTGAGTGCCTATC | Reverse transcription for miR-26a-1 |

Table S1-2 Oligonucleotide sequencess synthesized

| Names | Sequences |
|---|---|
| siACADM-571 | GGCCAGAAGAUGUGGAUAATT<br>UUAUCCACAUCUUCUGGCCTT |
| siACADM-900 | GGAUGAAGCUACCAAGUAUTT<br>AUACUUGGUAGCUUCAUCCTT |
| siACADM-1222 | GCACAAAUUCAAAGGAUUATT<br>UAAUCCUUUGAAUUUGUGCTT |
| siACADM-NC | UUCUCCGAACGUGUCACGUTT<br>ACGUGACACGUUCGGAGAATT |
| miR-26a-1-mimics | UUCAAGUAACCCAGGAUAGGCU |
| miR-26a-1-inhibitor | ACGGUAUCCUGGGUUACUUGAA |
| mimics-NC | UCACAACCUCCUAGAAAGAGUAGA |

| | |
|---|---|
| Inhibitor-NC | UCUACUCUUUCUAGGAGGUUGUGA |
